# Epidemiology and direct healthcare costs of Influenza-associated hospitalizations – nationwide inpatient data (Germany 2010-2019)

**DOI:** 10.1186/s12889-022-12505-5

**Published:** 2022-01-15

**Authors:** David Goettler, Patricia Niekler, Johannes G. Liese, Andrea Streng

**Affiliations:** Department of Pediatrics, University Hospital Würzburg, Josef-Schneider-Str. 2, 97080 Würzburg, Germany

**Keywords:** Influenza, Epidemiology, Healthcare costs, Hospitalization, ICD-10, Germany

## Abstract

**Introduction:**

Detailed and up-to-date data on the epidemiology and healthcare costs of Influenza are fundamental for public health decision-making. We analyzed inpatient data on Influenza-associated hospitalizations (IAH), selected complications and risk factors, and their related direct costs for Germany during ten consecutive years.

**Methods:**

We conducted a retrospective cost-of-illness study on patients with laboratory-confirmed IAH (ICD-10-GM code J09/J10 as primary diagnosis) by ICD-10-GM-based remote data query using the Hospital Statistics database of the German Federal Statistical Office. Clinical data and associated direct costs of hospital treatment are presented stratified by demographic and clinical variables.

**Results:**

Between January 2010 to December 2019, 156,097 persons were hospitalized due to laboratory-confirmed Influenza (J09/J10 primary diagnosis). The annual cumulative incidence was low in 2010, 2012 and 2014 (1.3 to 3.1 hospitalizations per 100,000 persons) and high in 2013 and 2015-2019 (12.6 to 60.3). Overall direct per patient hospitalization costs were mean (SD) 3521 EUR (± 8896) and median (IQR) 1805 EUR (1502; 2694), with the highest mean costs in 2010 (mean 8965 EUR ± 26,538) and the lowest costs in 2012 (mean 2588 EUR ± 6153). Mean costs were highest in 60-69 year olds, and in 50-59, 70-79 and 40-49 year olds; they were lowest in 10-19 year olds. Increased costs were associated with conditions such as diabetes (frequency 15.0%; 3.45-fold increase compared to those without diabetes), adiposity (3.3%; 2.09-fold increase) or immune disorders (5.6%; 1.88-fold increase) and with Influenza-associated complications such as Influenza pneumonia (24.3%; 1.95-fold), bacterial pneumonia (6.3%; 3.86-fold), ARDS (1.2%; 10.90-fold increase) or sepsis (2.3%; 8.30-fold). Estimated overall costs reported for the 10-year period were 549.6 Million euros (95% CI 542.7-556.4 million euros).

**Conclusion:**

We found that the economic burden of IAH in Germany is substantial, even when considering solely laboratory-confirmed IAH reported as primary diagnosis. The highest costs were found in the elderly, patients with certain underlying risk factors and patients who required advanced life support treatment, and median and mean costs showed considerable variations between single years. Furthermore, there was a relevant burden of disease in middle-aged adults, who are not covered by the current vaccination recommendations in Germany.

**Supplementary Information:**

The online version contains supplementary material available at 10.1186/s12889-022-12505-5.

## Background

Influenza disease is an acute viral infection of the respiratory tract caused by worldwide circulating influenza viruses group A (A[H1N1]pdm09 and A[H3N2]) and group B (B/Victoria lineage and B/Yamagata lineage) [1]. The Burden of Communicable Diseases in Europe study showed Influenza having the highest burden among all infectious diseases in Europe with 81.8 disability adjusted life years per 100,000 persons (95% UI: 76.9–86.5) [2]. Typical signs and symptoms are a sudden onset of fever, cough, runny nose, sore throat, headache, muscle and joint pain and feeling ill [1]. Though the level of evidence regarding risk factors for a severe course of disease is expandable, acknowledged high risk groups are infants (< 1 year of age), seniors (> 65 years of age), pregnant women and persons with comorbid illnesses [1, 3]. Serious complications potentially causing intensive care treatment and/or death include primary Influenza pneumonia, secondary bacterial pneumonia, neuromuscular complications and cardiac complications [3]. Common diagnostics are direct rapid antigen Influenza test, virus isolation and/or multiplex polymerase chain reaction of throat, nasal and nasopharyngeal samples [1, 4]. Based on initial test results and knowledge of the current epidemiologic and resistance situation treatment with neuraminidase-inhibitors is feasible in patients with severe disease or increased risk of severe disease while symptomatic treatment is advised in uncomplicated cases [1, 5].

Annual vaccination is usually recommended for health care workers, persons with high risk conditions and seniors but recommendations differ markedly between countries. In Germany, seasonal Influenza vaccination is currently recommended with quadrivalent inactivated Influenza vaccine (containing A[H1N1]pdm09, A[H3N2]), B/Victoria lineage and B/Yamagata lineage, respectively) with seasonal antigen combination as recommended by the World Health Organization for persons ≥ 60 years of age, pregnant women, residents in nursing homes, persons ≥ 6 month of age with high risk conditions (chronic cardiopulmonary disease, diabetes mellitus, chronic neurologic disorders, immune deficiency and immunosuppression) and persons with increased risk for transmission (health care workers, care givers to high risk persons); whereas seasonal Influenza vaccination in Germany is not generally recommended for persons < 60 years of age without neither high risk conditions nor increased risks for transmission [6]. In children, live attenuated Influenza vaccine may also be applied, as nasal spray.

The healthcare costs of Influenza disease as well as cost, efficacy, and safety of yearly immunization campaigns are aspects worth considering in the ongoing discussion of broadening the vaccine recommendation to healthy working adults, children and adolescents [7–9]. Therefore, country-specific economic burden estimates of Influenza disease are required [10]. The German Influenza surveillance mechanisms include estimates of Influenza-associated excess medical visits, excess hospitalizations, Influenza-associated work days lost, need for care and death, but detailed data on direct medical costs stratified by age groups, underlying conditions and complications are not publicly available [11]. In the present analyses, we close this data gap by contributing inpatient data on Influenza-associated hospitalizations, selected complications and risk factors for severe disease, and related costs of the entire German population during ten consecutive years.

## Methods

### Study design

We conducted a retrospective observational cost-of-illness study using the database of the German Federal Statistical Office (DeStatis) on German Hospital Statistics [12]. This database provides the statistical basis for Public Health-related reporting, planning and governmental decision making. It comprises hospital discharge diagnoses coded according to the International Classification of Diseases 10th edition, German modification (ICD-10-GM) [13]. Primary diagnosis and secondary diagnoses are distinguished [14]. According to the German Coding Guidelines the primary diagnosis at hospital discharge represents the diagnosis that, after having considered all relevant information of the hospital stay, had been responsible for hospital admission. Secondary diagnoses comprise relevant comorbidities and/or complications. Diagnostic and therapeutic measures conducted during hospital treatment are available and coded through Operation and Procedure Codes (OPS) [15]. Annual reporting to the Federal Statistical Office, including main and secondary diagnosis, OPS codes, elementary demographic data, outcome and related inpatient costs, is mandatory for all hospitals in Germany, who need to document these data for health insurance claims. Data from German Hospital Statistics are available for academic institutions on reasonable request by remote data query. To guarantee anonymity of patients, data analysis resulting in case numbers per at least one associated subgroup less than 3 persons (i.e. ≤ 2 persons) are censored by the Statistical Office. Incidence per season was calculated as the number of patients with a J09/J10 primary diagnosis hospitalized that year divided by the total population of Germany that year, expressed per 100,000 persons.

### Study population and case selection

The entire German population serves as study population. The database was screened for persons of all age groups discharged after an in-hospital treatment between 01 January 2010 and 31 December 2019 (discharge date) due to laboratory-confirmed Influenza as defined by a primary diagnosis of J09 (“Influenza due to identified zoonotic or pandemic influenza virus”, generally used for coding Influenza A(H1N1)pdm09) or J10 (“Influenza due to identified seasonal Influenza virus”) and these patients were included in the present analysis. ICD-10-GM code J11 ("Seasonal Influenza, virus not identified") as primary diagnosis is subject to confounding with non-Influenza respiratory viruses and was excluded, as were all patients with any Influenza code reported solely as secondary diagnosis.

Patients hospitalized for less than 24 h are counted as inpatients with a hospital length of stay of one day. Multiple hospitalizations of the same individual, the diagnostic approach of virus identification and the Influenza vaccination status are not recorded or not traceable in the present cohort.

Patients with certain risk factors for severe Influenza and patients with well-known complications were selected according to ICD-10-GM codes reported as any secondary diagnoses for our analysis (Table 1). Selected risk factors included Pregnancy, Diabetes, Adiposity, and Immune Disorder. Selected complications included Bronchitis caused by Influenza, Pneumonia caused by Influenza, Pneumonia caused by another virus, Pneumonia caused by bacteria, Myocarditis, Encephalitis, Acute respiratory distress syndrome (ARDS), Sepsis, and Otitis Media.

**Table 1:** Relevant ICD-10-GM and Operation and Procedure (OPS) codes with their respective characterization

Code	Categorization
**ICD-10-GM Code**	**Influenza Codes**
J09	Influenza due to identified zoonotic or pandemic Influenza virus
J10	Influenza due to identified seasonal Influenza virus
J10.0	Influenza with pneumonia, seasonal Influenza virus identified
J10.1 J10.8	Influenza with other respiratory manifestations, seasonal Influenza virus identified Influenza with other manifestations, seasonal Influenza virus identified
J11	Influenza, seasonal Influenza virus not identified
J11.0	Influenza with pneumonia, seasonal Influenza virus not identified
J11.1	Influenza with other respiratory manifestations, seasonal Influenza virus not identified
J11.8	Influenza with other manifestations, seasonal Influenza virus not identified
U69.20	Influenza A/H1N1 pandemic 2009
	**Risk factors for a severe course of Influenza (selection)**
O00-99	Pregnancy
E10-14	Diabetes
E66	Adiposity
C00-97 **or** B20-24 **or** D80-90 **or** Z94	Immune disorder
	**Complications of Influenza (selection)**
J20.8 **or** J20.9 **or** J21.8 **or** J21.9 **or** J22	Bronchitis, Influenza^a^
J10.0 **or** J11.0	Pneumonia, Influenza^a^
J12	Pneumonia, other viral
J13-18	Pneumonia, bacterial
**OPS-Code**	**Treatment codes (selection)**
8-980 **or** 8-98d **or** 8-98f	Intensive care treatment
8-852	ECMO (Extracorporeal Membrane Oxygenation)
8-8521	Pre-ECMO
8-8522	PECLA (Pumpless arterio-venous extracorporeal lung assist)
8-8523	Minimized heart-lung machine
8-8525	Veno-venous extracorporeal CO2 removal
8-8526	RA-PA-ECMO-module right heart support

These codes were used to define groups and subgroups for the present analyses on Influenza-associated hospitalizations, derived from the database on German Hospital Statistics from the Research Data Centers of the German Federal Statistical Office, Germany, 2010-2019. ^a^Unspecified codes for bronchitis or pneumonia in combination with an Influenza-specific primary diagnosis (J09/J10).

### Study outcome

The cumulative annual hospitalization rate per 100,000 persons due to laboratory-confirmed Influenza in Germany is assessed. Clinical burden of diseases caused by or associated with Influenza virus are presented by indicator variables and stratified by age group. Direct medical costs per inpatient Influenza case are presented at the hospital perspective stratified by demographic data, relevant risk factors and selected Influenza-associated complications. Non-reimbursed direct medical costs (e.g. additional charges, out-of-pocket payments), direct non-medical costs (e.g. transportation, rehabilitation, and housekeeping assistance as a consequence of illness) and indirect costs (work or school absenteeism, burden to caregivers, short-term productivity loss, early retirement or death due to the illness) are not represented. Costs were presented in 2019 euros using the German Consumer Price index [16].

### Statistical analyses

The cumulative annual hospitalization rate per 100,000 persons is calculated with exact Poisson 95% confidence intervals and stratified by age group. German national census data serve as denominator. Nominal and ordinal data are reported as number of persons (percent). P-values are calculated using asymptotic Chi-Squared test. Continuous data is reported as mean (standard deviation) and median with interquartile range (IQR, 25th percentile to 75th percentile). Using IBM SPSS Statistics Version 26 (IBM Corporation, One New Orchard Road, Armonk, New York, USA), p-values are calculated using Mann-Whitney U-test or Kruskal-Wallis H-test, as appropriate. We used bootstrap samples – with 1000 replicates of the same size of the original dataset and sampled with replacement – to estimate the mean per patient costs using SAS® Version 9.4 (SAS Institute Inc., Cary, NC, USA).

## Results

### Influenza-associated hospitalizations

Between January 2010 and December 2019, 156,097 patients were hospitalized due to a laboratory-confirmed Influenza infection with a primary diagnosis of J09 (N= 25,226) or J10 (N= 131,989). The median age was 56 years (IQR 9; 77) and the mean age was 47.2 years (SD ± 32.5). Out of 156,097 patients hospitalized, 46.9% were in the >59 years age group (N= 73,286) and females accounted for 48.4% (N= 75,609) of all cases. Overall, J10 was the most commonly reported ICD-10-GM code (N= 131,989, 84.6%), especially in the >59 years age group (88.8% of all 73,286 cases). The 18-59 years age group had the largest proportion of the Influenza A/H1N1 pandemic 2009 strain, with J09 ICD-10-GM diagnosis (22.8%) and U69.20 ICD-10-GM diagnosis (9.2%) (Table 2).

### Selected risk factors and complications

Diabetes was the most frequently reported selected risk factor (N= 23,465, 15.0%), with the >59 years age group having the largest proportion (27.7%). In the study year 2017, 20% (N= 4,144) of patients hospitalized with a J09/J10 primary diagnosis in Germany were reported to be diabetic, the highest percentage in all study years (Additional file 1). Immune disorders were reported in 5.6% (N= 8,791) of all cases. In 2014, when 49.4% of all cases were patients <18 years old, immune disorders were seen at the largest proportion in patients <18 years old (7.1%), compared to all other study years (Additional file 1). From the selected complications, Pneumonia caused by the Influenza virus was the most often reported by all cases (N= 37,898, 24.3%), especially for the >59 years age group (N= 24,260, 33.1%) (Table 2). From the complications reported as secondary diagnoses, sepsis (N= 3,590) was reported as the most frequent, in 2.3% of the cases and was most frequent in the 18-59 years age group (N= 1,133, 3.3%) and the >59 age group (N=2,345, 3.2%). Otitis Media (N= 2,909, 1.9%), on the other hand, was most frequently reported among children, <18 years of age (N= 2,644, 5.5%).

### Treatment and Outcomes

In total, the hospitals reported 6.1% of all cases (N= 9,513) having visited the intensive care unit (ICU) while hospitalized, with the majority of patients in the >59 years age group (N= 5,849). However, the 18-59 years age group had the largest percentage of patients (8.4%, N=2,918) that had to be treated at the ICU. Only 1.6% (N=746) in the <18 years age group were reported visiting the ICU, and less than 0.1% in this age group were reported having any ECMO (Extracorporeal Membrane Oxygenation), Pre-ECMO, PECLA (Pumpless arterio-venous extracorporeal lung assist), Minimized heart-lung machine, or Veno-venous extracorporeal CO2 removal treatment. On the other hand, 361 (1.0%) of patients in the 18-59 years age group had an ECMO procedure done while hospitalized with influenza-related illness. Total number that had to undergo Pre-ECMO, PECLA, Minimized heart-lung machine, and Veno-venous extracorporeal CO2 removal treatments were very low, therefore the information is not included in Table 2 to protect the privacy of the patients. In total, 5,411 (3.5%) patients hospitalized with a J09 or J10 primary diagnosis had a fatal outcome, with older adults (>59 years) comprising 86.9% of the fatal cases (N=4,700). Mortality in older adults (>59 years) was 6.4%, while children (<18 years) had the lowest mortality (0.2%, N= 86). The median number of hospital days for all patients was 5 days (IQR 3; 7), with patients in the >59 age group having the longest stays (median = 7, IQR 4; 10) (Table 2).

**Table 2:** General characteristics of study patients with Influenza-associated hospitalization in Germany, January 2010 - December 2019

	Age, years				
	**All**	**<18**	**18-59**	**>59**	**p-value**^a^
All cases, N (%)	156,097 (100)	47,982 (100)	34,829 (100)	73,286 (100)	
Age, median (IQR)	56 (9; 77)	3 (1; 7)	46 (33; 54)	78 (70; 84)	<0.001
Age, mean (±SD)	47.2 (± 32.5)	4.8 (± 4.8)	42.9 (± 12.2)	77.1 (± 8.9)	<0.001
**Sex**					
Female, N (%)	75,609 (48.4)	21,752 (45.3)	16,868 (48.4)	36,989 (50.5)	<0.001
**Selected Risk Factors, N(%)**					
Pregnancy	423 (0.3)	7 (0.0)	416 (1.2)	0 (0.0)	<0.001
Diabetes	23,465 (15.0)	148 (0.3)	3,046 (8.7)	20,271 (27.7)	<0.001
Adiposity	5,089 (3.3)	197 (0.4)	1,755 (5.0)	3,137 (4.3)	<0.001
Immune disorder	8,791 (5.6)	582 (1.2)	2,875 (8.3)	5,334 (7.3)	<0.001
**Influenza-associated ICD-10-GM codes, N(%)**					
Influenza due to identified zoonotic or pandemic Influenza virus (J09)	25,226 (16.2)	8,443 (17.6)	7,947 (22.8)	8,836 (12.1)	<0.001
Influenza due to identified seasonal Influenza virus (J10)	131,989 (84.6)	39,879 (83.1)	27,217 (78.1)	64,893 (88.5)	<0.001
Influenza with pneumonia, seasonal Influenza virus identified (J10.0)	37,811 (24.2)	6,691 (13.9)	6,885 (19.8)	24,235 (33.1)	<0.001
Influenza with other respiratory manifestations, seasonal Influenza virus identified (J10.1)	79,765 (51.1)	27,953 (58.3)	16,936 (48.6)	34,876 (47.6)	<0.001
Influenza with other manifestations, seasonal Influenza virus identified (J10.8)	15,516 (9.9)	5,477 (11.4)	3,588 (10.3)	6,451 (8.8)	<0.001
Influenza, virus not identified (J11)^b^	889 (0.6)	225 (0.5)	243 (0.7)	421 (0.6)	<0.001
Influenza with pneumonia, seasonal Iinfluenza virus not identified (J11.0)^b^	176 (0.1)	35 (0.1)	59 (0.2)	82 (0.1)	<0.001
Influenza with other respiratory manifestations, seasonal Influenza virus not identified (J11.1)^b^	611 (0.4)	151 (0.3)	159 (0.5)	301 (0.4)	0.003
Influenza with other manifestations, seasonal Influenza virus not identified (J11.8)^b^	107 (0.1)	40 (0.1)	27 (0.1)	40 (0.1)	0.133
Influenza A/H1N1 pandemic 2009 (U69.20)^b^	8,160 (5.2)	2,835 (5.9)	3,219 (9.2)	2,106 (2.9)	<0.001
**Selected Complications, N(%)**					
Bronchitis, Influenza	5,982 (3.8)	1,575 (3.3)	1,272 (3.7)	3,135 (4.3)	<0.001
Pneumonia, Influenza	37,898 (24.3)	6,712 (14.0)	6,926 (19.9)	24,260 (33.1)	<0.001
Pneumonia, other viral	476 (0.3)	186 (0.4)	126 (0.4)	164 (0.2)	<0.001
Pneumonia, bacterial	9,773 (6.3)	1,277 (2.7)	2,551 (7.3)	5,945 (8.1)	<0.001
Myocarditis	383 (0.2)	57 (0.1)	173 (0.5)	153 (0.2)	<0.001
Encephalitis	224 (0.1)	104 (0.2)	65 (0.2)	55 (0.1)	<0.001
ARDS	1,889 (1.2)	92 (0.2)	1,004 (2.9)	793 (1.1)	<0.001
Sepsis	3,590 (2.3)	112 (0.2)	1,133 (3.3)	2,345 (3.2)	<0.001
Otitis Media	2,909 (1.9)	2,644 (5.5)	160 (0.5)	105 (0.1)	<0.001
**Treatment/Outcome, N(%)**					
Intensive care	9,513 (6.1)	746 (1.6)	2,918 (8.4)	5,849 (8.0)	<0.001
ECMO	540 (0.3)	19 (0.0)	361 (1.0)	160 (0.2)	<0.001
Pre-ECMO	36 (0.0)	XXX	XXX	XXX	
PECLA	49 (0.0)	XXX	XXX	XXX	
Minimized heart-lung machine	50 (0.0)	XXX	XXX	XXX	
Veno-venous extracorporeal CO2 removal	5 (0.0)	XXX	XXX	XXX	
Fatality	5,411 (3.5)	86 (0.2)	625 (1.8)	4,700 (6.4)	<0.001
Hospital stay in days, median (IQR)	5 (3; 7)	3 (2; 4)	4 (2; 4)	7 (4; 10)	<0.001

Data are N (percent) or median (quartiles), unless otherwise specified. Cases were assigned to study years (January - December) by date of hospital discharge. Sex is unknown for 5 of 156,097 persons and these persons were included as female. ICD-10-GM codes are either primary diagnosis (PD) or secondary diagnosis (SD); multiple nominations of influenza codes per patient are possible. If at least one age group contained less than 3 patients, data was removed for patient data protection and marked XXX. ARDS: Acute respiratory distress syndrome. ECMO: Extracorporeal membrane oxygenation. PECLA: Pumpless arterio-venous extracorporeal lung assist. ^a^Kruskal-Wallis or Mann-Whitney test. ^b^In the present analyses, any diagnoses J11 or U69.20 were only recorded as secondary diagnoses.

### Incidence

**Fig. 1:**
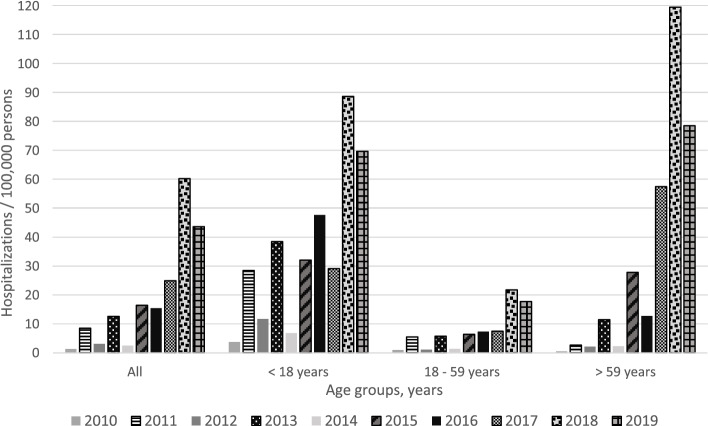
Hospitalizations per 100,000 persons estimated by 156,097 persons in Germany hospitalized with laboratory-confirmed Influenza. Between January 2010 and December 2019, stratified by study year and age group.

The peak of Influenza-related hospitalizations for the ten-year span was observed Jan – Dec 2018 across all three main age groups (total incidence: 60.3). The year with the lowest incidence was 2010 (total incidence: 1.3) (Fig. 1). Patients <18 years of age exhibited the highest incidence of all age groups from 2010 to 2016. However, the >59 years age group experienced the highest incidence rate from 2017 to 2019. Exact incidence values can be found in Additional file 2.

### Direct hospitalization costs per patient

The arithmetic mean (SD) hospitalization costs per Influenza patient from 2010 to 2019 in Germany was €3521 (±€8886), while the median (IQR) per patient cost was €1805 (€1502; €2694) (Table 3 and Additional file 3). The total extrapolated cost for all IAH patients between January 2010 and December 2019 was 549,213,935.50 EUR (95% CI 542.7-556.4 million euros). Male patients incurred a higher mean and median direct cost than female patients in total and every individual year (Additional file 3). Overall, hospitalized patients in the >59 years age group had the highest per patient mean (€4162, SD±€9469) and median (€2561, IQR €1627; €2743) cost. More specifically, patients in the 60-69 years age group experienced the highest direct mean cost (€5434, SD±€13,574) and those in the ≥90 age group incurred the highest direct median cost (€2688, IQR €1722; €2858).

High mean costs were also observed in the age groups 50-59 years, 70-79 years and 40-49 years. The lowest per patient cost was observed in children 10-19 years old (median= €1504, IQR €1366; €2258 / mean= €2145 SD±€5255). Patients with diabetes, adiposity, and immune disorders had a significantly higher overall per patient cost: 3.45 fold, 2.09 fold, and 1.88 fold increase in cost than those without such underlying conditions respectively (p<0.001 for all). The exception is patients who were pregnant, which actually incurred a final cost that was lower than the average (median= €1627, IQR €1419; €2420 / mean= €3277 SD±€16,178). From the selected complications, patients with ARDS incurred the highest median and mean costs (median= €29,015, IQR €12,337; €54,781 / mean= €38,372 SD±€35,996), greatly surpassing the direct costs of any other complication in Table 3, however they make up 1.2% (N=1,889) of the total 156,097 patients. The second most costly selected complication is sepsis (N=3590), with a mean (SD) per patient cost of €29,215 (±€36,080). The least expensive selected complication, Bronchitis caused by Influenza (N= 5,982), had a mean (SD) cost of €3384 (±€9910) per patient. Our results show that patients with pneumonia caused by Influenza (1.95 fold increase), bacterial pneumonia (3.86 fold increase), Sepsis (8.30 fold increase), and ARDS (10.90 fold increase) as complications had a higher cost than those without such complications (p<0.001 for all). Patients that had to receive care at the ICU had a significantly higher per patient median cost of €11,983 (IQR €4,086; €28,733; p<0.001) than non-ICU patients and those who had an ECMO procedure had the highest per patient mean and median cost (mean= €57,290 SD±€48,551; median= €45,622, IQR €29,299; €74,159; p<0.001). Fatal cases incurred a mean cost of €13,917 (SD±€22,188).

**Table 3:** Direct per patient hospitalization costs^a^ of persons hospitalized with laboratory-confirmed Influenza in Germany, January 2010 - December 2019

	N (%)	Median € (IQR)**	Mean € (±SD)
**All**	156,097 (100)	1805 (1502; 2694)	3521 (±8886)
**Sex**			
Female	75,609 (48.4)	1765 (1501; 2690)	3277 (±7903)
Male	80,488 (51.6)	1809 (1504; 2696)	3751 (±9714)
**Age, years**			
<18	47,982 (30.7)	1644 (1466; 2405)	2208 (±4526)
18-59	34,829 (22.3)	1698 (1470; 2688)	3982 (±11,559)
>59	73,286 (46.9)	2561 (1627; 2743)	4162 (±9469)
**Age, years**			
<10	39,388 (25.2)	1663 (1501; 2445)	2230 (±4374)
10-19	9,717 (6.2)	1504 (1366; 2258)	2145 (±5255)
20-29	5,426 (3.5)	1629 (1370; 2384)	2542 (±7917)
30-39	6,409 (4.1)	1631 (1430; 2610)	2965 (±7378)
40-49	8,262 (5.3)	1724 (1472; 2688)	4084 (±11,867)
50-59	13,609 (8.7)	1809 (1501; 2735)	5101 (±14,149)
60-69	16,777 (10.7)	2291 (1506; 2741)	5434 (±13,574)
70-79	25,627 (16.4)	2408 (1578; 2741)	4275 (±9912)
80-89	25,009 (16.0)	2613 (1629; 2771)	3489 (±5952)
≥90	5,873 (3.8)	2688 (1722; 2858)	2903 (±2230)
**Selected Risk Factor**			
Pregnancy	423 (0.3)	1627 (1419; 2420)	3277 (±16,178)
Diabetes	23,465 (15.0)	2632 (1629; 3183)	5231 (±12,150)
Adiposity	5,089 (3.0)	2613 (1627; 3635)	7344 (±16,520)
Immune disorder	8,791 (5.6)	2696 (1632; 5019)	6604 (±16,582)
**Selected Complications**			
Bronchitis, Influenza	5,982 (3.8)	1850 (1506; 2690)	3384 (±9910)
Pneumonia, Influenza	37,898 (24.3)	2735 (2688; 4316)	6872 (±14,104)
Pneumonia, other viral	476 (0.3)	3786 (2645; 15,354)	16,328 (±27,631)
Pneumonia, bacterial	9,773 (6.3)	3499 (2683; 11,572)	13,582 (±25,076)
Myocarditis	383 (0.2)	2623 (1759; 3890)	5730 (±10,598)
Encephalitis	224 (0.1)	2752 (1782; 9653)	12,505 (±22,905)
ARDS	1,889 (1.2)	29,015 (12,337; 54,781)	38,372 (±35,996)
Sepsis	3,590 (2.3)	15,818 (4454; 41,009)	29,215 (±36,080)
Otitis Media	2,909 (1.9)	1726 (1520; 2530)	2308 (±4624)
**Treatment/Outcome**			
Intensive Care	9,513 (6.1)	11,983 (4086; 28,733)	22,365 (±28,346)
ECMO	540 (0.3)	45,622 (29,299; 74,159)	57,290 (±48,551)
Fatality	5,411 (3.5)	4153 (2685; 16,114)	13,917 (±22,188)

^a^standardized to 2019 EUR. Data are N (percent) or median (quartiles), unless otherwise specified. Cases were assigned to study years (January - December) by date of hospital discharge. Sex is unknown for 5 of 156,097 persons. These persons were included as female. ICD-10-GM codes are primary diagnosis or secondary diagnosis if not otherwise indicated. ARDS: Acute respiratory distress syndrome. ECMO: Extracorporeal membrane oxygenation. Differences in (median) costs among subgroups were tested for significance; for each selected risk factor, complication and treatment/outcome, the costs in patients with a specific condition were compared with the costs in patients without this specific condition. P-values (after Kruskal-Wallis or Mann-Whitney tests were performed) were all highly significant (p<0.001)

### Annual economic burden of hospitalizations associated with Influenza

Study year 2015 had the highest median (IQR) per patient cost, €2584 (€1419; €2636) while study year 2010 had the highest mean (SD) cost, €8965 (±€26,538) (Table 4). A bootstrapping analysis of the mean total cost per episode resulted in a 95% confidence interval of 3477-3565 €. Although 2018 boasted the greatest number of Influenza-related hospitalizations (N= 50,073, 32.1%), the median per patient cost (€1763, IQR €1504; €2696) were below that of the total for the 10 years included in the study. For all study years, patients who were >59 years old incurred the highest hospitalization costs. In addition, those with a J09/J10 main diagnosis and J09/J10 as any secondary diagnosis were reported to have had a considerably more costly stay, followed by those with just a J09 primary diagnosis (Additional file 3).

**Table 4:** Annual direct per patient hospitalization costs^a^ of persons hospitalized with laboratory-confirmed Influenza in Germany, January 2010 - December 2019

	N (%)	Median € (IQR)		Mean € (±SD)		(95% CI)	
**All**	156,097 (100)	1805 (1502; 2694)		3521 (±8886)		3521 (3477-3565)	
**Study year**							
2010	1,087 (0.7)	2551 (2469; 3924)		8965 (±26,538)			
2011	6,864 (4.4)	2291 (1441; 2352)		3849 (±9781)			
2012	2,534 (1.6)	1600 (1366; 2405)		2588 (±6153)			
2013	10,202 (6.5)	2348 (1334; 2432)		3806 (±9283)			
2014	2,052 (1.3)	2516 (1392; 2578)		3886 (±8405)			
2015	13,521 (8.7)	2584 (1419; 2636)		3556 (±9013)			
2016	12,750 (8.2)	1697 (1433; 2621)		3842 (±11,231)			
2017	20,680 (13.2)	1762 (1470; 2690)		3145 (±6308)			
2018	50,073 (32.1)	1763 (1504; 2696)		3535 (±8926)			
2019	36,334 (23.3)	1731 (1629; 2739)		3342 (±7827)			
^a^standardized to 2019 EUR.							

## Discussion

### Burden of disease

Our study, during the years 2010-2019, analyzes 156,097 reported patients hospitalized in Germany because of a laboratory confirmed Influenza primary diagnosis. Overall, the majority of patients were >59 years (46.9%) and had a J10 ("Influenza due to identified seasonal influenza virus") primary diagnosis (84.6%). Among patients >59 years, 27.7% of those hospitalized had diabetes as a pre-existing condition and 33.1% suffered from pneumonia caused by Iinfluenza as a complication. The youngest (<18 years) and oldest (>59 years) age groups carried the highest burden of Influenza measured by the incidence rates. Out of all the risk factors we selected to evaluate, diabetes was the most common in all years except in 2011, where immune disorder was more common. This can be explained by the fact that patients >59 years (the largest represented group in all years except 2011) have a greater instance of diabetes, but in 2011, the <18 years age group accounted for the majority of the hospitalized patients (54.6%). A study from Von der Beck et al. (2017), also based on the Database of the German Federal Statistical Office, analyzed data of Influenza inpatients from the year 2005 to 2012 [17]. Von der Beck found that younger patients were more frequently hospitalized during the 2009/2010 A(H1N1) influenza pandemic. Our data from 2010 and the post-pandemic years shows the remnants of that pandemic in 2010-2013, where patients <18 years comprised of more than 45% of all Influenza related hospitalizations in Germany. Compared to our study, Von der Beck et al. concluded that very young (0-4 years) and very old (>60 years) patients are less frequently hospitalized during the pandemic, whereas during non-pandemic seasons these age groups bear the highest burden of disease. Our results show that, since 2017, patients >59 years are the majority again and make up more than 50% of all Influenza-associated hospitalizations. The greatest burden of disease regarding frequency of hospitalizations has in the last couple of years, thus, shifted from children (5-14 years) [17] to older adults (>59 years).

As already observed by Von der Beck, interestingly, a substantial burden also occurred in adults 18-59 years during the pandemic. During the post-pandemic years, they also showed considerable proportions of IAH, with over 30% during the years 2011 and 2014, and usually representing between 20 and 30% of the annual IAH. In the present analysis, adults of this age group have the largest proportion of serious complications such as ARDS (2.9%, N= 1004) and Sepsis (3.3%, N= 1133). Adults (18-59 years) also have the highest proportion of patients that had to receive intensive care treatment (8.4%, N= 2918) and invasive ECMO therapy (1.0%, N= 361). This is particularly relevant since adults in this age group fall outside of the recommendations of seasonal Influenza vaccinations in Germany.

### Costs

The current analysis describes and quantifies the direct healthcare costs of laboratory confirmed Influenza hospitalizations in Germany from January 2010 to December 2019. The overall mean (SD) annual per patient direct cost of hospitalization was 3521€ (±8886€) while the overall median (IQR) annual per patient cost was 1805€ (1502€; 2694€). In the span of ten years, 156,097 hospitalized patients with a confirmed Influenza infection (ICD-10-GM J09/J10 primary diagnosis) amounted an estimated 549,213,936 EUR in just direct costs.

Patients incurred higher costs when intensive care treatment and/or a complication, especially ARDS, Sepsis, and Pneumonia caused by a virus other than Influenza, was reported. These results correspond with those found by a Karve et al. study that found that healthcare costs among Influenza patients with complications double those of Influenza patients without any, although in our results in some cases with complications the cost was almost 10 times higher than the average median and mean costs for all Influenza hospitalized patients [18].

Our study includes patients hospitalized due to a laboratory confirmed Influenza diagnosis, that is with the ICD-10-GM code J09 or J10 reported as primary diagnosis. According to Ehlken et al. 2015, over 92% of Influenza attributable episodes are classified as “Influenza, virus not identified” J11 [19]. In our study, we explicitly excluded patients solely with a primary diagnosis of J11 in order to avoid including patients hospitalized with Influenza-like symptoms that could be caused by a number of other viruses, some of which might have different risk factors and complications associated with them.

The 2009/2010 Influenza A(H1N1) pandemic claimed the lives of 151,700-575,400 people worldwide during that influenza season [20]. It is estimated that roughly 80% of all A(H1N1)pdm09-related deaths occurred in people younger than 65 years, which was a shift from the typical influenza mortality rates, which disproportionately affect people older than 65 years. Influenza A(H1N1)pdm09 is only associated with the ICD-10-GM J09 code. Our individual year analysis (Additional Table 2) reveals that in 2010-2011, the pandemic strain was still the most prevalently coded in hospitalized Influenza patients, but in 2012 there is a sudden shift and the proportion of patients with J09 gradually begins to decrease (with reasonable fluctuations) until dropping below 10% in 2018. This decrease in the use of the Influenza code J09 is likely to reflect a change in coding procedures, as the previous ‘pandemic’ Influenza virus A(H1N1)pdm09 is now considered a ‘seasonal’ virus and may therefore be coded increasingly as “J10”. It is noticeable that in 2010, compared to the ten-year average, the total number of persons hospitalized was low (as the main wave of the pandemic occurred in 2009 and, hence was not covered by the present analysis), while mean costs per person were high (Table 4). This may be due to higher prevalence of J09 (Additional Table 2), higher costs associated with J09 (Additional Table 3), and higher prevalence of ECMO treatment (data not shown) in that study year.

Comparison with other studies is difficult due to differences in healthcare system, unit costs of applied resources, case definition, and study population. Our study includes costs that are reported by the hospitals and not estimated. In a study conducted by Haas et al. on 65,826 Influenza patients in a health claims data analysis from the season 2012/2013, it was estimated that in Germany mean Influenza hospitalization costs were roughly 5832€ which is much higher than in our study (3806€) [21]. An explanation for this may be that the Haas et al. study defines influenza hospitalization through the ICD-10-GM codes J09, J10 and J11 either as primary or as any secondary diagnosis. It is possible that relevant chronic conditions or complications coded as primary diagnosis with an influenza code as secondary diagnosis were associated with higher costs than those with a J09/J10 primary diagnosis with the chronic condition or complication stated as secondary diagnosis. In contrast, the estimated average inpatient Influenza case cost between 2012 and 2014 in a further recent analysis of German health claims data by Scholz et al. (for cost calculations reference year 2014) based on Influenza as primary diagnosis was 2033€ (SD±2952€), lower than the costs we calculated for those years [22]. However, their data set contained only 458 hospitalized patients and the case definition included J11 as primary diagnosis ("Influenza, virus not identified") which may be associated with less costs.

Similar to a US study by Young-Xu et al. we found the highest per patient costs in the age groups in the 40-79 age range [23]. Generally, patients older than 90 years old did not incur particularly high costs in our cohort. This is probably because of the high risk and high-cost invasive procedures such as ECMO are less frequently used in very elderly patients. In fact, our study reveals that Influenza patients who had an ECMO procedure while hospitalized incurred costs that were more than 10 times the overall median and mean per patient cost, even though they made up less than 0.5% of all patients.

In our study, patients >59 years have the longest average length of hospital stay (7 days). According to the CDC, older adults tend to have longer stay at the hospital due to complications associated with underlying diseases [24]. We also see in our study that patients in the >59 years age group have higher rates of complications. Sepsis was reported in 3.2% (N= 2,345) of all cases >59 years, whereby patients with a Sepsis complications have a mean (SD) per patient cost of 29,215€ (±36,080€). Sepsis (N= 1,133, 3.3%) and ARDS (N= 1,004, 2.9%) were also reported in 18-59 year olds more frequently than in the other two age groups, the latter having the highest per patient mean (SD) cost out of all the selected complications (38,372€, ±35,996€). We also see that the 18-59 years age group have the largest proportion of patients receiving treatment at the ICU (8.4%) and having ECMO procedures (1.0%) performed. ECMO is a bypass circuit that essentially serves as an artificial membrane lung with patients with severe respiratory complications. Complications on ECMO are not uncommon and the procedure is generally considered a high-risk operation that is reserved for patients with very severe respiratory complications, such as ARDS. Higher rates of ECMO treatment among the 18-59 years age group could be an indication that they are subject to more severe complications or that physicians are more hesitant to use high-risk procedures on children (<18 years) and older adults (>59 years). Nevertheless, ECMO use is still a rare treatment option for hospitalized Influenza patients.

Our results show that substantial burden and costs occur in adults >59 years: they make up 46.9% of all hospitalized Influenza patients and have the overall highest median and mean per patient direct cost. In Germany, the Robert Koch Institute recommends that all adults 60 years of age or older should receive yearly Influenza vaccination [24, 25]. Improving Influenza vaccination uptake may have the potential to reduce this burden [26]. As of 2017, less than 40% of the population older than 59 years of age were vaccinated against the seasonal flu, and that percentage was a slight decrease from the previous year. This is well below the European Union’s target of 75% vaccination coverage [27]. It is now more apparent than ever that in Germany, Influenza vaccine recommendations for adults should be revised in the future and public health efforts in the country need to increase, especially among the elderly population and those with a chronic underlying disease.

Our analysis closely resembles a cost of illness study, where the goal is to quantify the cost, or economic burden, of a specific condition. Cost of illness studies have ultimately a descriptive role and are not a true economic evaluation because there is no analysis of the competing course of action [28]. Therefore, although we are able to extrapolate mean per patient direct costs, ultimately, we are not able to appraise whether these costs are significantly high or low enough to create a negative effect on the economy. However, since we cover rare events with serious economic consequences, cost data (and the annual fluctuation in costs) may contribute information for public health decision making.

It should be noted that our study was focused on direct claim costs for patients. A 2007 study in the US reported that indirect costs (such as productivity losses) were about 10 times higher than direct medical costs [29]. Hence, the total healthcare costs of IAH are most likely far higher.

#### Limitations

There are some important limitations to the interpretation of our results. Our study only includes inpatient data, so symptomatic cases who do not seek medical attention or seek outpatient care are not considered for analysis. Also, no out-of-pocket payments or co-payments that are not reimbursed by the German sickness funds are part of our analysis. A study by Molinari et al. in 2007 showed that the actual costs of seasonal Influenza is grossly underestimated since the disease is so underreported [12]. This is not ideal since, as mentioned previously, various studies have reported that direct inpatient medical costs are only a small portion of the total economic burden of disease compared to indirect costs such as work absenteeism. In addition, we have no information on the vaccination status of the patients, so we are not able to see whether the vaccine had any protective effect against the more severe complications, and thereby the cost. Although our study has great external validity since the whole German population is included, there is low internal validity due to potential coding issues. However, to characterize Influenza patients we focused in the present study on the Influenza ICD-10-GM codes used for laboratory-confirmed Influenza, leaving out the code J11 ("Influenza, not laboratory confirmed"). We had previously conducted a sensitivity analysis that showed that J11 as main diagnosis represented only a small proportion of cases (17% of patients with a main diagnosis J09/J10/J11) and had only low impact on the costs.

Furthermore, we focused on only those patients with laboratory-confirmed Influenza codes used as primary diagnosis. Hence, it is likely that the ‘true’ number of hospitalized patients with Influenza is far higher, as e.g. those patients were missed with a primary diagnosis of a likely Influenza-associated complication, e.g. secondary bacterial pneumonia, or myocarditis. Thus, our cost analysis restricted to well-defined IAHs represents only a minimum estimate of the true costs.

## Conclusions

This retrospective analysis showed German inpatient clinical data and associated direct medical costs of laboratory-confirmed Influenza based on ICD-10-GM codes in ten consecutive years. We found that the economic burden of IAH in Germany is substantial, even when considering solely laboratory-confirmed IAH. The highest costs were found in the elderly, patients with certain underlying risk factors and patients who required advanced life support treatment, and median and mean costs showed considerable variations between single years. Furthermore, there was a relevant burden of disease in middle-aged adults, who are not covered by the current vaccination recommendations in Germany.

## Supplementary Information

**Additional file 1**

**Additional file 2**

**Additional file 3**

## Data Availability

The data sources for the analyses of this study are available from the Research Data Center (RDC) of the Federal Statistical Office and Statistical Offices of the Länder (Germany), DRG-Statistik 2010-2019, doi: 10.21242/23141.2010.00.00.1.1.0 to 10.21242/23141.2019.00.00.1.1.0; however, restrictions apply to the availability of these data, which were used under license for the current study (own calculations; project 3730-2018). Hence, the source data, the analysis programs and the resultant data extractions are not publicly available.

## Abbreviations

ARDS: Acute Respiratory Distress Syndrome
CDC: Centers for Disease Control
ECMO: Extracorporeal Membrane Oxygenation
IAH: Influenza-Associated Hospitalization
ICD-10-GM: 10th revision of the International Statistical Classification of Diseases and Related Health Problems (German Modification)
OPS: Operational and Procedure Codes
PECLA: Pumpless arterio-venous extracorporeal lung assist
RKI: Robert Koch Institute
