# Supplementary material for: Epidemiology and direct healthcare costs of Influenza-associated hospitalizations – nationwide inpatient data (Germany 2010-2019)
Source: BMC Public Health. 2022 Jan 15;22:108. doi: 10.1186/s12889-022-12505-5 (PMC8761049; doi:10.1186/s12889-022-12505-5)
Supplement: Supplementary file 1 — Additional file 1 [file 12889_2022_12505_MOESM1_ESM.pdf]

Additional Table 1 Descriptive data of 156,097 persons hospitalized with Influenza (J09/J10 primary diagnosis) in Germany between Jan 2010 and Dec 2019, stratified by study year.

		2010	2011	2012	2013	2014	2015	2016	2017	2018	2019
All, N(%)		1087 (100)	6864 (100)	2534 (100)	10202 (100)	2052 (100)	13521 (100)	12750 (100)	20680 (100)	50073 (100)	36334 (100)
Age, N(%)	<18	502 (46.2)	3751 (54.6)	1537 (60.7)	5036 (49.4)	900 (43.9)	4278 (31.6)	6432 (50.4)	3943 (19.1)	12068 (24.1)	9535 (26.2)
	18-59	468 (43.1)	2535 (36.9)	521 (20.6)	2644 (25.9)	640 (31.2)	2974 (22.0)	3410 (26.7)	3460 (16.7)	10039 (20.0)	8138 (22.4)
	>59	117 (10.8)	578 (8.4)	476 (18.8)	2522 (24.7)	512 (25.0)	6269 (46.4)	2908 (22.8)	13277 (64.2)	27966 (55.9)	18661 (51.4)
Age, years, median (IQR)	<18	3 (1; 10)	4 (1; 9)	4 (1; 8)	3 (1; 8)	3 (1; 6)	3 (1; 8)	4 (1; 8)	3 (1; 10)	3 (1; 7)	3 (1; 7)
	18-59	40 (27; 51)	39 (28; 50)	39 (30; 50)	45 (34; 53)	43 (30; 52)	45 (33; 53)	40 (29; 51)	48 (33; 55)	48 (37; 55)	46 (33; 54)
	>59	69 (63; 74)	69 (63; 74)	76 (69; 82)	74 (67; 81)	74 (67; 79)	78 (72; 84)	74 (67; 79)	79 (73; 85)	78 (70; 83)	78 (70; 84)
Age, < 1 month		3 (0.3)	41 (0.6)	6 (0.2)	35 (0.3)	11 (0.5)	48 (0.4)	48 (0.4)	44 (0.2)	118 (0.2)	90 (0.2)
Female sex[a]		520 (47.8)	3127 (45.6)	1161 (45.8)	4775 (46.8)	941 (45.9)	6351 (47.0)	5781 (45.3)	10258 (49.6)	25116 (50.2)	17579 (48.4)
**Influenza code, N(%)**											
Influenza due to identified zoonotic or pandemic influenza virus (J09)		704 (64.8)	4106 (59.8)	634 (25.0)	3424 (33.6)	661 (32.2)	4110 (30.4)	2324 (18.2)	3113 (15.1)	3009 (6.0)	3141 (8.6)
Influenza due to identified seasonal influenza virus (J10)		414 (38.1)	2896 (42.2)	1910 (75.4)	6917 (67.8)	1407 (68.6)	9569 (70.8)	10523 (82.5)	17676 (85.5)	47287 (94.4)	33390 (91.9)
Influenza with pneumonia, seasonal influenza virus identified (J10.0)		221 (20.3)	903 (13.2)	559 (22.1)	2076 (20.3)	455 (22.2)	3062 (22.6)	2646 (20.8)	5168 (25.0)	13402 (26.8)	9319 (25.6)
Influenza with other respiratory manifestations, seasonal influenza virus identified (J10.1)		142 (13.1)	1843 (26.9)	1217 (48)	4391 (43.0)	823 (40.1)	5959 (44.1)	6522 (51.2)	10816 (52.3)	27162 (54.2)	20890 (57.5)
Influenza with other manifestations, seasonal influenza virus identified (J10.8)		58 (5.3)	169 (2.5)	146 (5.8)	497 (4.9)	135 (6.6)	623 (4.6)	1441 (11.3)	1810 (8.8)	7182 (14.3)	3455 (9.5)
Influenza, virus not identified (J11)		10 (0.9)	60 (0.9)	13 (0.5)	65 (0.6)	12 (0.6)	77 (0.6)	80 (0.6)	117 (0.6)	280 (0.6)	175 (0.5)
Influenza with pneumonia, seasonal influenza virus not identified (J11.0)		3 (0.3)	20 (0.3)	XXX (XXX)	15 (0.1)	XXX (XXX)	18 (0.1)	24 (0.2)	28 (0.1)	38 (0.1)	28 (0.1)
Influenza with other respiratory manifestations, seasonal influenza virus not identified (J11.1)		7 (0.6)	31 (0.5)	10 (0.4)	44 (0.4)	10 (0.5)	53 (0.4)	43 (0.3)	80 (0.4)	208 (0.4)	125 (0.3)
Influenza A/H1N1 pandemic 2009 (U69.20)		269 (24.7)	2361 (34.4)	104 (4.1)	1347 (13.2)	174 (8.5)	729 (5.4)	1043 (8.2)	276 (1.3)	917 (1.8)	940 (2.6)
**Selected Risk Factors, N(%)**											
Pregnancy		18 (1.7)	45 (0.7)	7 (0.3)	30 (0.3)	XXX (XXX)	43 (0.3)	38 (0.3)	32 (0.2)	120 (0.2)	89 (0.2)
Diabetes		81 (7.5)	363 (5.3)	151 (6.0)	909 (8.9)	190 (9.3)	2034 (15.0)	974 (7.6)	4144 (20.0)	8682 (17.3)	5937 (16.3)
Adiposity		43 (4.0)	166 (2.4)	36 (1.4)	263 (2.6)	66 (3.2)	452 (3.3)	343 (2.7)	688 (3.3)	1704 (3.4)	1328 (3.7)
Immune disorder		73 (6.7)	410 (6.0)	106 (4.2)	634 (6.2)	146 (7.1)	898 (6.6)	603 (4.7)	1169 (5.7)	2862 (5.7)	1890 (5.2)
**Selected Complications, N(%)**											
Bronchitis, Influenza		58 (5.3)	278 (4.1)	83 (3.3)	383 (3.8)	73 (3.6)	548 (4.1)	414 (3.2)	821 (4.0)	2042 (4.1)	1282 (3.5)
Pneumonia, Influenza		224 (20.6)	921 (13.4)	559 (22.1)	2087 (20.5)	456 (22.2)	3073 (22.7)	2654 (20.8)	5182 (25.1)	13416 (26.8)	9326 (25.7)
Pneumonia, other viral		20 (1.8)	51 (0.7)	10 (0.4)	57 (0.6)	13 (0.6)	48 (0.4)	75 (0.6)	41 (0.2)	99 (0.2)	62 (0.2)
Pneumonia, bacterial		213 (19.6)	741 (10.8)	111 (4.4)	818 (8.0)	165 (8.0)	922 (6.8)	837 (6.6)	1193 (5.8)	2813 (5.6)	1960 (5.4)
**Treatment/Outcome, N(%)**											
Intensive care		162 (14.9)	524 (7.6)	79 (3.1)	753 (7.4)	155 (7.6)	800 (5.9)	861 (6.8)	1166 (5.6)	2925 (5.8)	2088 (5.7)
Extracorporeal circulation		32 (2.9)	51 (0.7)	XXX (XXX)	75 (0.7)	15 (0.7)	39 (0.3)	70 (0.5)	29 (0.1)	134 (0.3)	94 (0.3)
Hospital stay, days		5 (3; 10)	4 (2; 6)	4 (2; 6)	4 (3; 7)	4 (2; 7)	5 (3; 8)	4 (2; 6)	5 (3; 8)	5 (3; 8)	5 (3; 7)
Fatality		50 (4.6)	133 (1.9)	26 (1.0)	273 (2.7)	38 (1.9)	437 (3.2)	238 (1.9)	813 (3.9)	2164 (4.3)	1239 (3.4)

Data are n (percent) or median (quartiles), unless otherwise specified, and cases were assigned to study years (January - December) by date of hospital discharge. **a** Sex is unknown for 5 of 156,097 persons. These persons are included as female. Continuous variables values are presented as Median (IQR). All patients had a primary diagnosis of J09 or J10.
