# Supplementary material for: Epidemiology and direct healthcare costs of Influenza-associated hospitalizations – nationwide inpatient data (Germany 2010-2019)
Source: BMC Public Health. 2022 Jan 15;22:108. doi: 10.1186/s12889-022-12505-5 (PMC8761049; doi:10.1186/s12889-022-12505-5)
Supplement: Supplementary file 2 — Additional file 2 [file 12889_2022_12505_MOESM2_ESM.pdf]

Additional Table 2 Hospitalizations per 100,000 persons estimated by 156,097 persons in Germany hospitalized with laboratory-confirmed Influenza (J09/J10 primary diagnosis) between January 2010 and December 2019, stratified by study year (January – December) and age group.

	Study Year									
**Age groups**	**2010**	**2011**	**2012**	**2013**	**2014**	**2015**	**2016**	**2017**	**2018**	**2019**
All	1,3	8,5	3,1	12,6	2,5	16,5	15,5	25	60,3	43,7
< 18 years	3,8	28,5	11,7	38,5	6,9	32,1	47,7	29,1	88,8	69,7
18 - 59 years	1	5,5	1,1	5,8	1,4	6,4	7,4	7,5	21,8	17,8
> 59 years	0,5	2,7	2,2	11,5	2,3	27,9	12,8	57,5	119,6	78,6
