# Supplementary material for: Epidemiology and direct healthcare costs of Influenza-associated hospitalizations – nationwide inpatient data (Germany 2010-2019)
Source: BMC Public Health. 2022 Jan 15;22:108. doi: 10.1186/s12889-022-12505-5 (PMC8761049; doi:10.1186/s12889-022-12505-5)
Supplement: Supplementary file 3 — Additional file 3 [file 12889_2022_12505_MOESM3_ESM.pdf]

Additional Table 3: Direct per patient hospitalization costs (standardized to 2019 EUR) of 156,097 persons hospitalized with Influenza (J09/J10 primary diagnosis) in Germany between January 2010 and December 2019, stratified by year, rounded to the nearest Euro.

		**2010**		**2011**		**2012**		**2013**	
		Median € (IQR)	Mean € (±SD)	Median € (IQR)	Mean € (±SD)	Median € (IQR)	Mean € (±SD)	Median € (IQR)	Mean € (±SD)
All		2551 (2469; 3924)	8965 (±26538)	2291 (1441; 2352)	3849 (±9781)	1600 (1366; 2405)	2588 (±6153)	2348 (1334; 2432)	3806 (±9283)
Age, years	<18	2520 (1881; 2581)	3404 (±6615)	2274 (1376; 2342)	2429 (±4853)	1505 (1359; 2341)	2123 (±4652)	1554 (1334; 2367)	2168 (±4248)
	18-59	2581 (2513; 5610)	13003 (±36687)	2297 (2171; 2370)	5100 (±12864)	2305 (1359; 2405)	2394 (±3875)	2353 (1334; 2432)	5249 (±12295)
	>59	4027 (2535; 11675)	16760 (±27052)	2352 (2278; 4224)	7592 (±14984)	2393 (1366; 3640)	4300 (±10574)	2408 (1608; 3852)	5567 (±11988)
Age, years	<10	2522 (2369; 2581)	3094 (±4596)	2278 (1441; 2352)	2483 (±4184)	1505 (1366; 2345)	2019 (±3046)	1556 (1348; 2367)	2178 (±4058)
	10-19	2520 (1587; 2598)	4461 (±10747)	2258 (1337; 2297)	2353 (±6597)	1366 (1328; 2331)	2510 (±8255)	1348 (1303; 2351)	2165 (±4998)
	20-29	2535 (2196; 2718)	7197 (±15417)	2297 (1376; 2352)	3256 (±10269)	1366 (1309; 2405)	1763 (±1144)	2348 (1312; 2408)	3672 (±8351)
	30-39	2561 (2338; 3586)	8595 (±16950)	2297 (1376; 2352)	4159 (±9574)	1883 (1359; 2393)	2076 (±2035)	2347 (1312; 2408)	3756 (±9406)
	40-49	2598 (2522; 5965)	11796 (±21850)	2330 (2274; 2468)	5759 (±13379)	2338 (1359; 2405)	2461 (±2355)	2353 (1334; 2432)	5121 (±12128)
	50-59	3969 (2535; 14126)	22732 (±60490)	2342 (2291; 3925)	7400 (±16748)	2345 (1366; 2515)	3223 (±6827)	2367 (1348; 3786)	6976 (±15001)
	60-69	4344 (2551; 25939)	19447 (±29505)	2352 (2297; 4563)	9150 (±17423)	2393 (1366; 3611)	4918 (±14812)	2408 (1557; 3890)	7407 (±16715)
	70-79	3978 (2522; 25651)	17463 (±27073)	2352 (2278; 3971)	6489 (±12691)	2368 (1366; 2659)	4235 (±10093)	2408 (1611; 3821)	5423 (±10843)
	80-89	XXX (XXX)	XXX (XXX)	XXX (XXX)	XXX (XXX)	2405 (1842; 3749)	4077 (±6577)	2408 (1842; 3852)	4008 (±6103)
	>90	XXX (XXX)	XXX (XXX)	XXX (XXX)	XXX (XXX)	2428 (1627; 3749)	2816 (±1123)	2432 (2059; 3786)	3053 (±1874)
Age, years	<1	2552 (2498; 2622)	3530 (±4868)	2461 (1488; 2527)	3153 (±7206)	1529 (1497; 2481)	2319 (±2915)	2425 (1473; 2487)	2454 (±4015)
	1-4	2513 (2369; 2581)	2888 (±3002)	2278 (1488; 2330)	2444 (±3274)	1561 (1366; 2345)	2110 (±3810)	1848 (1437; 2367)	2096 (±3318)
	5-9	2495 (1405; 2535)	3052 (±6712)	2258(1340; 2297)	2152 (±2647)	1366 (1326; 2305)	1701 (±899)	1348 (1303; 2348)	2144 (±5243)
	10-14	2469 (1565; 2551)	3265 (±9333)	1962 (1337; 2297)	2242 (±5191)	1366 (1326; 2305)	2921 (±10547)	1348 (1303; 2351)	2198 (±5669)
	15-17	2522 (2419; 2598)	5628 (±11384)	2258 (1340; 2297)	2313 (±8639)	1366 (1331; 2331)	1931 (±2575)	1334 (1303; 2351)	1967 (±2964)
Sex [a]	Male	2551 (2469; 3988)	10239 (±33057)	2297 (1449; 2352)	4044 (±10404)	1604 (1366; 2405)	2631 (±6422)	2348 (1348; 2432)	3957 (±9667)
	Female	2551 (2369; 3395)	7579 (±16724)	2291 (1376; 2352)	3617 (±8976)	1600 (1359; 2405)	2537 (±5821)	2338 (1334; 2432)	3633 (±8824)
Influenza Code	J09	2581 (2522; 3978)	9898 (±30482)	2314 (2287; 2448)	4427 (±11307)	2405 (2335; 2405)	2648 (±2608)	2408 (2351; 2533)	4713 (±10981)
	J10	2369 (1405; 2598)	6064 (±17228)	1441 (1340; 2287)	2694 (±6045)	1468 (1359; 2331)	2535 (±6807)	1473 (1312; 2378)	3148 (±7369)
	J09 and J10**	23531 (2611; 40038)	25137 (±23052)	2361 (2297; 9605)	10333 (±16597)	2468 (XXX;XXX)	8848 (±19085)	3161 (2351; 15179)	14458 (±26055)

Cases were assigned to study years (January - December) by date of hospital discharge. [a] Sex is unknown for 5 of 156,097 persons. These persons are included as female. All patients had a primary diagnosis of J09 or J10. **J09/J10 as any secondary diagnosis in addition to J09/J10 as a primary diagnosis

		2014		2015		2016		2017	
		Median € (IQR)	Mean € (±SD)	Median € (IQR)	Mean € (±SD)	Median € (IQR)	Mean € (±SD)	Median € (IQR)	Mean € (±SD)
All		2516 (1392; 2578)	3686 (±8405)	2584 (1419; 2636)	3556 (±8885)	1697 (1433; 2621)	3842 (±11231)	1762 (1470; 2690)	3145 (±6308)
Age, years	<18	1675 (1392; 2574)	2452 (±5711)	1587 (1391; 2584)	2268 (±5347)	1573 (1433; 2607)	2395 (±6455)	1611 (1468; 1814)	1956 (±2216)
	18-59	2517 (1390; 2578)	4126 (±8898)	2055 (1394; 2636)	3720 (±10681)	1715 (1433; 2621)	4518 (±12976)	1472 (1469; 2690)	2864 (±6316)
	>59	2574 (1675; 3017)	5309 (±11027)	2613 (1667; 2748)	4357 (±9742)	2621 (1447; 2912)	6251 (±15893)	2683 (1470; 2694)	3572 (±7036)
Age, years	<10	1713 (1392; 2574)	2445 (±5302)	1654 (1419; 2584)	2362 (±5879)	1590 (1433; 2617)	2454 (±6547)	1611 (1470; 2110)	2024 (±2336)
	10-19	1626 (1359; 2517)	2412 (±6811)	1419 (1391; 2584)	1902 (±2060)	1433 (1433; 1697)	2141 (±5842)	1470 (1466; 1722)	1881 (±2932)
	20-29	2513 (1390; 2574)	2597 (±4628)	1653 (1391; 2613)	2998 (±15017)	1447 (1433; 2617)	2112 (±4587)	1470 (805; 1762)	1766 (±1308)
	30-39	1675 (1390; 2574)	2595 (±3963)	1646 (1391; 2613)	2897 (±8495)	1514 (1433; 2621)	2895 (±7606)	1472 (1468; 2685)	2843 (±6018)
	40-49	2537 (1663; 2578)	4139 (±7675)	2305 (1407; 2636)	3456 (±7680)	2246 (1433; 2648)	5363 (±16244)	1472 (1469; 2690)	2610 (±4471)
	50-59	2537 (1392; 2684)	6142 (±12891)	2584 (1407; 2636)	4802 (±11618)	2621 (1447; 2882)	7271 (±17218)	1717 (1469; 2690)	3372 (±7887)
	60-69	2574 (1675; 3690)	7092 (±14695)	2584 (1419; 2800)	6177 (±14755)	2621 (1447; 3985)	8283 (±19848)	1855 (1470; 2690)	4218 (±10502)
	70-79	2537 (1626; 2590)	4795 (±10068)	2584 (1419; 2636)	4259 (±9331)	2617 (1447; 2648)	6118 (±16089)	2631 (1470; 2694)	3656 (±7365)
	80-89	2574 (2517; 3638)	4268 (±6463	2613 (1700; 2825)	3781 (±7239)	2617 (1697; 2882)	3678 (±5595)	2683 (1472; 2694)	3369 (±5396)
	>90	2578 (1713; 2756)	2442 (±832)	2613 (2218; 2825)	2857 (±1839)	2621 (1794; 2858)	2725 (±1684)	2690 (1755; 2835)	2960 (±2652)
Age, years	<1	1622 (1533; 2705)	2711 (±3928)	1654 (1556; 2617)	2433 (±4905)	1697 (1573; 2580)	2941 (±7803)	1613 (1611; 2679)	2217 (±2274)
	1-4	1787 (1520; 2574)	2566 (±6693)	1675 (1549; 2584)	2400 (±6541)	1590 (1447; 2621)	2619 (±7509)	1611 (1470; 2344)	2003 (±2340)
	5-9	1562 (1359; 2517)	1882 (±901)	1419 (1391; 2584)	2211 (±5026)	1433 (1433; 1715)	1923 (±3369)	1470 (1466; 1722)	1873 (±2378)
	10-14	1675 (1359; 2517)	2993 (±10222)	1419 (1391; 2584)	1908 (±2176)	1433 (1433; 1697)	2193 (±6019)	1470 (1466; 1722)	1822 (±2044)
	15-17	1666 (1359; 2517)	1953 (±1482)	1419 (1391; 2584)	1872 (±2088)	1433 (1430; 1697)	2065 (±6068)	1470 (1466; 1722)	1667 (±1418)
Sex [a]	Male	2517 (1392; 2778)	3903 (±9368)	2584 (1419; 2636)	3905 (±9903)	1697 (1433; 2621)	3921 (±11130)	2045 (1470; 2690)	3325 (±6873)
	Female	2516 (1392; 2574)	3431 (±7098)	2573 (1419; 2636)	3162 (±7555)	1697 (1433; 2621)	3746 (±11351)	1760 (1470; 2690)	2962 (±5671)
Influenza Code	J09	2574 (2517; 2578)	4721 (±10337)	2613 (2584; 2636)	3647 (±8260)	2621 (2621; 2648)	5321 (±12942)	2690 (2683; 2694)	3515 (±5548)
	J10	1626 (1390; 2574)	3118 (±7118)	1656 (1407; 2613)	3439 (±8868)	1573 (1433; 2615)	3466 (±10707)	1722 (1470; 2690)	3067 (±6405)
	J09 and J10**	3884 (2578; 10851)	11342 (±15321)	2613 (2584; 3635)	8227 (±18553)	2621 (2621; 8008)	10267 (±17602)	2690 (2049; 4087)	5532 (±8937)

Cases were assigned to study years (January - December) by date of hospital discharge. [a] Sex is unknown for 5 of 156,097 persons. These persons are included as female.
All patients had a primary diagnosis of J09 or J10. **J09/J10 as any secondary diagnosis in addition to J09/J10 as a primary diagnosis.

		2018		2019	
		Median € (IQR)	Mean € (±SD)	Median € (IQR)	Mean € (±SD)
All		1763 (1504; 2696)	3535 (±8926)	1731 (1629;2739)	3342 (±7827)
Age, years	<18	1642 (1501; 2042)	2166 (±4273)	1727 (1629;1812)	2076 (±2975)
	18-59	1506 (1501; 2688)	3708 (±11013)	1631 (1627;2736)	3483 (±10014)
	>59	2409 (1505; 2722)	4065 (±9496)	2253 (1630;2743)	3927 (±8360)
Age, years	<10	1645 (1504; 2444)	2186 (±4203)	1728 (1629;1876)	2078 (±2548)
	10-19	1504 (1501; 1760)	2094 (±5006)	1629 (1627;1807)	2071 (±5005)
	20-29	1505 (873; 1765)	2092 (±4106)	1630 (922;1809)	2217 (±7333)
	30-39	1505 (1501; 2685)	2664 (±7066)	1631 (1627;2735)	2539 (±5004)
	40-49	1506 (1501; 2688)	3746 (±12586)	1631 (1627;2735)	3478 (±10809)
	50-59	1759 (1501; 2696)	4539 (±12429)	1645 (1629;2739)	4445 (±12009)
	60-69	1810 (1505; 2696)	5056 (±13228)	1937 (1629;2741)	4874 (±11450)
	70-79	2115 (1505; 2716)	4285 (±10216)	2240 (1629;2743)	4019 (±9084)
	80-89	2420 (1506; 2752)	3472 (±6257)	2562 (1631;2743)	3419 (±5390)
	>90	2688 (1760; 2967)	2891 (±2022)	2735 (1631;2743)	2895 (±2356)
Age, years	<1	1646 (1641; 2647)	2434 (±4405)	1730 (1726;2802)	2261 (±2693)
	1-4	1645 (1505; 2688)	2147 (±3735)	1728 (1629;2149)	2055 (±2390)
	5-9	1505 (1501; 1760)	2065 (±4964)	1631 (1627;1809)	1967 (±2764)
	10-14	1504 (1501; 1760)	2041 (±4215)	1630 (1627;1807)	2245 (±6306)
	15-17	1504 (1501; 1760)	2080 (±5231)	1629 (1627;1807)	1777 (±2146)
Sex [a]	Male	1765 (1505; 2696)	3802 (±9781)	1801 (1629;2741)	3556 (±8695)
	Female	1760 (1504; 2696)	3270 (±7978)	1730 (1629;2739)	3112 (±6774)
Influenza Code	J09	2696 (2688; 2719)	4478 (±9773)	2741 (2735;2743)	3824 (±9148)
	J10	1760 (1504; 2694)	3466 (±8811)	1726 (1629;2739)	3281 (±7601)
	J09 and J10**	2696 (1809; 3249)	6450 (±17161)	2741 (1730;3855)	6253 (±17452)

Cases were assigned to study years (January - December) by date of hospital discharge. [a] Sex is unknown for 5 of 156,097 persons. These persons are included as female. All patients had a primary diagnosis of J09 or J10. **J09/J10 as any secondary diagnosis in addition to J09/J10 as a primary diagnosis
